# Dynamic Interhemispheric Desynchronization in Marmosets and Humans With Disorders of the Corpus Callosum

**DOI:** 10.3389/fncir.2020.612595

**Published:** 2020-12-21

**Authors:** Diego Szczupak, Cecil C. Yen, Cirong Liu, Xiaoguang Tian, Roberto Lent, Fernanda Tovar-Moll, Afonso C. Silva, in collaboration with the IRC5 Consortium

**Affiliations:** ^1^Department of Neurobiology, University Pittsburgh Brain Institute, University of Pittsburgh, Pittsburgh, PA, United States; ^2^Cerebral Microcirculation Section, Laboratory of Functional and Molecular Imaging, National Institute of Neurological Disorders and Stroke, National Institutes of Health, Bethesda, MD, United States; ^3^Institute of Neuroscience, CAS Center for Excellence in Brain Science and Intelligence Technology, Chinese Academy of Sciences, Shanghai, China; ^4^Instituto de Ciências Biomédicas, Universidade Federal do Rio de Janeiro, Rio de Janeiro, Brazil; ^5^Instituto D’Or de Pesquisa e Ensino, Rio de Janeiro, Brazil

**Keywords:** cingulate cortex, corpus callosum, dynamic functional connectivity, dysgenesis of the corpus callosum, marmosets, non-human primates, quasi-periodic patterns, sensory cortex

## Abstract

The corpus callosum, the principal structural avenue for interhemispheric neuronal communication, controls the brain’s lateralization. Developmental malformations of the corpus callosum (CCD) can lead to learning and intellectual disabilities. Currently, there is no clear explanation for these symptoms. Here, we used resting-state functional MRI (rsfMRI) to evaluate the dynamic resting-state functional connectivity (rsFC) in both the cingulate cortex (CG) and the sensory areas (S1, S2, A1) in three marmosets (*Callithrix jacchus*) with spontaneous CCD. We also performed rsfMRI in 10 CCD human subjects (six hypoplasic and four agenesic). We observed no differences in the strength of rsFC between homotopic CG and sensory areas in both species when comparing them to healthy controls. However, in CCD marmosets, we found lower strength of quasi-periodic patterns (QPP) correlation in the posterior interhemispheric sensory areas. We also found a significant lag of interhemispheric communication in the medial CG, suggesting asynchrony between the two hemispheres. Correspondingly, in human subjects, we found that the CG of acallosal subjects had a higher QPP correlation than controls. In comparison, hypoplasic subjects had a lower QPP correlation and a delay of 1.6 s in the sensory regions. These results show that CCD affects the interhemispheric synchrony of both CG and sensory areas and that, in both species, its impact on cortical communication varies along the CC development gradient. Our study shines a light on how CCD misconnects homotopic regions and opens a line of research to explain the causes of the symptoms exhibited by CCD patients and how to mitigate them.

## Introduction

The corpus callosum is the primary white matter structure that connects the brain’s hemispheres in a predominantly homotopic fashion (Hofer and Frahm, 2006). Developmental malformations (dysgenesis) of the corpus callosum (CCD) have a high prevalence of 1.47:10,000 (Ballardini et al., 2018). They can lead to cognitive deficits that resemble those found in autism spectrum disorders (Paul et al., 2014) and many other conditions featuring cognitive impairments (Brown and Paul, 2019).

The presence of CCD causes the brain to rewire and form several alternative white-matter fiber bundles that are generally not present in healthy brains. These alternative pathways include the Probst bundle (Probst, 1901; Tovar-Moll et al., 2007), the sigmoid bundle (Paul et al., 2007; Tovar-Moll et al., 2007), and inter-cortical bundles (Tovar-Moll et al., 2014). Although diffusion MRI data shows that the brain hemispheres in CCD patients are disconnected (Owen et al., 2013a; Jakab et al., 2015), resting-state functional MRI (rsfMRI) shows that CCD brains have a bilateral representation of resting-state networks (Tyszka et al., 2011), as well as normal network-based-statistics (Owen et al., 2013a).

To better understand how CCD brains can rewire to compensate for callosal deficits and process interhemispheric information, animal models are needed. Many genetically modified mice lines have been described to feature CCD, but most do not survive until adulthood. Also, they do not present hypoplasia or partial CCD, only complete agenesis (Ren et al., 2007; Fothergill et al., 2014). Other spontaneous mouse strains, such as the Balb/c (Wahlsten et al., 2003), can produce different CCD phenotypes with varying callosal malformations. However, accessing cognitive behavior in mice is challenging.

Because they are phylogenetically closer to humans and exhibit complex cognitive functions and social behavior (Miller et al., 2016), non-human primate (NHP)s are the ideal model of CCD. One great example is the common marmoset (*Callithrix jacchus*), a highly social NHP that has several practical advantages in biomedical research, such as small size, easy adaptation to life in captivity, no zoonotic transmission, and prolific breeding (Miller et al., 2016; Silva, 2017). Marmosets exhibit a highly sophisticated repertoire of social behavior, including cooperative rearing of infants, foraging for food and vigilance against predators, social grooming, scent-marking behavior, gaze following, and antiphonal calling (Miller et al., 2016). Many of these behaviors are likely to be disrupted by developmental brain malformations like CCD. And yet, to the best of our knowledge, there have been no reports of spontaneous or induced CCD in marmosets.

In this work, we used anatomical MRI to screen a population of 38 marmosets for callosal malformations. We found four animals with spontaneous hypoplasia of the CC. We then used resting-state fMRI to characterize their interhemispheric resting-state functional connectivity (rsFC), focusing our investigation on the cingulate cortex and the sensory areas, which are known to recruit interhemispheric callosal connections. We also performed analogous experiments in 10 CCD human subjects (six hypoplasic and four agenesic). In both marmosets and humans, we found that CCD affects the interhemispheric synchrony of cortical communication with varying impacts along the CC development gradient. The present results bring in a new understanding of how CCD misconnects homotopic regions and opens a line of research to explain the causes of the symptoms exhibited by CCD patients and how to mitigate them.

## Materials and Methods

### Marmosets

We complied with all relevant ethical regulations for animal testing and research. All procedures in this study were approved by the Animal Care and Use Committee of the National Institute of Neurological Disorders and Stroke. In this study, 38 adult common marmosets of both sexes (23 males and 15 females) age varying from 4 to 12 years and weight 380 g to 650 g were housed either as two adults of the same sex per cage or as a single breeding pair with pre-weaned young in cages furnished with hammocks, plastic tubes, and plush toys for environmental enrichment. The light cycle was 12 h light:12 h dark.

For obtaining structural T1-weighted MRI of their heads, the animals were sedated with 10 mg/kg ketamine and 0.054 mg/kg atropine, orally intubated and anesthetized with 2% isoflurane. Vital signs (body temperature, heart rate, arterial oxygen saturation, and end-tidal CO_2_) were continuously monitored and recorded every 15 min for the entire duration of the MRI session. At the end of the imaging session, the animals were fully recovered from anesthesia before they were returned to their cages.

For obtaining rsfMRI data, the marmosets were acclimated to remaining still in the MRI scanner under fully conscious, awake conditions following the acclimatization protocol previously published (Silva et al., 2011). Anatomical flexible helmets were designed to fit the animal’s head perfectly and provide a robust and comfortable frame to minimize head motions during the scan. We monitored the animal’s face with an in-bore camera during scans to assure that the animal was awake and comfortable throughout the imaging.

#### MRI Acquisition

All marmoset MRI experiments were performed on a 7 T, 300 mm horizontal magnet interfaced with a Bruker AVANCE AVIII console (Bruker Corporation, RRID:SCR_017365; Bruker, Billerica, MA, USA). The MRI system was equipped with a 150 mm gradient set capable of generating 450 mTm^−1^ within 150 μs (Resonance Research Inc., Billerica, MA, USA). We used an in-house 11 cm ID linear birdcage RF coil for excitation and an eight-element phased-array receive-only RF coil for signal detection.

##### T_1_-Weighted MRI

We acquired a T1-weighted image of 32 anesthetized animals using a three-dimensional MPRAGE sequence with the following parameters: TR = 6,000 ms, TE = 2.8 ms, TI = 1,200 ms, FOV = 28 × 36 mm, matrix size = 112 × 144 × 96 yielding a isotropic resolution of 250 μm, number of averages = 1, acquisition time = 10 min. This scan provided the necessary CNR to delineate the CC. We also analyzed MRI data from six deceased animals with an *ex vivo* T2* weighted sequence (Luciano et al., 2016) that provided enough CNR to delineate the CC.

##### Awake Resting-State fMRI

We acquired resting-state fMRI of 10 conscious, awake marmosets (seven controls and three CCD) using an in-house 10-channel phased-array coil that yielded greater SNR (Liu et al., 2019), allowing accurate brain network analysis. Resting-state fMRI data was acquired using a multi-slice 2D gradient-echo EPI sequence: TR = 2,000 ms, TE = 22.2 ms, flip angle = 70.4°, FOV = 28 × 36 mm, matrix size = 56 × 72, 38 axial slices, resolution = 0.5 mm isotropic, number of repetitions = 1,512 volumes/run, each run is 17 min long. We collected six consecutive runs from all 10 animals (total acquisition time 1 h 42 min).

### Human Subjects

For this study, we selected 10 CCD human subjects (six males, four females) with ages ranging from 2 to 33 years (Table 1). Six patients had CC hypoplasia (HP), where the CC is whole but smaller in the midline section area, and the remaining four had CC agenesis (AC), where there is no remnant of the CC. An experienced neuroradiologist confirmed the patients’ diagnosis. All procedures were consented to and approved by the Ethics Committee of our institution.

**Table 1:** Demographic human data.

Subject	Age	CCD	Sex
366	9	AC	M
659	9	AC	M
662	33	AC	F
704	2	AC	F
706	10	AC	F
708	20	AC	M
661	7	HP	M
711	21	HP	F
718	21	HP	M
723	22	HP	M
LS4025	25–35	CT	M
LS4036	25–35	CT	M
LS4041	25–35	CT	F
LS4043	25–35	CT	F
LS4047	25–35	CT	F

*CCD, dysgenesis of the corpus callosum; AC, agenesic; HP, hypoplasic; CT, control*.

#### fMRI Acquisition

All human MRI experiments were performed on a Siemens 3T PRISMA scanner (Siemens AG Medical Solutions, Erlangen, Germany). Resting-state fMRI data were acquired following the human connectome project (HCP) protocol (Van Essen et al., 2013), with a TR = 800 ms, TE = 37 ms, flip angle = 52, FOV = 93.6 × 93.6 mm, slice = 2 mm, yielding an isotropic spatial resolution of 2 mm and a temporal resolution of 0.8 s and compared to five HCP Lifespan control subjects. The rsfMRI data were acquired in a single session and comprised a minimum of 420 and a maximum of 500 time-points per subject. Subjects strongly affected by CCD symptoms (four AC and two HP) were sedated for the entire duration of the rsfMRI session.

### Data Processing

#### rsfMRI Data Processing

Images were top-up corrected in FSL (FSL, RRID:SCR_002823, Andersson and Sotiropoulos, 2016), aligned in space, despiked, dropped first 5 TRs, temporally filtered for 1 to 0.1 Hz bandpass, spatially blurred (1 mm for marmosets and 4 mm for humans), and motion censored in AFNI (Analysis of Functional NeuroImages, RRID:SCR_005927) software (Cox, 1996). No ICA denoising algorithms or global signal regression were used in this work.

In marmosets, we manually drew ROIs to select, bilaterally, the anterior (aCC) and posterior (pCC) cingulate cortex and the sensory areas S1, S2, and A1 as representative cortical regions that are known to be connected *via* the CC. ROIs were manually drawn at three anteroposterior levels: the anterior commissure (anterior), the anterior thalamus (medial), and the posterior thalamus (posterior) level. The cingulate cortex ROIs were defined as the medial supracallosal cortex. The sensory cortex was selected as the supracallosal cortical area lateral to the cingulate bundle (for example, see Figure 3). We asserted that the ROIs had the same size and were symmetrically placed in both hemispheres. The rationale for choosing these planes was that at the anterior level, the animals have the highest variability in CC thickness; at the medial level, the animals have the thinnest CC section; and at the posterior level, the CC starts to thicken again.

**Figure 1:**
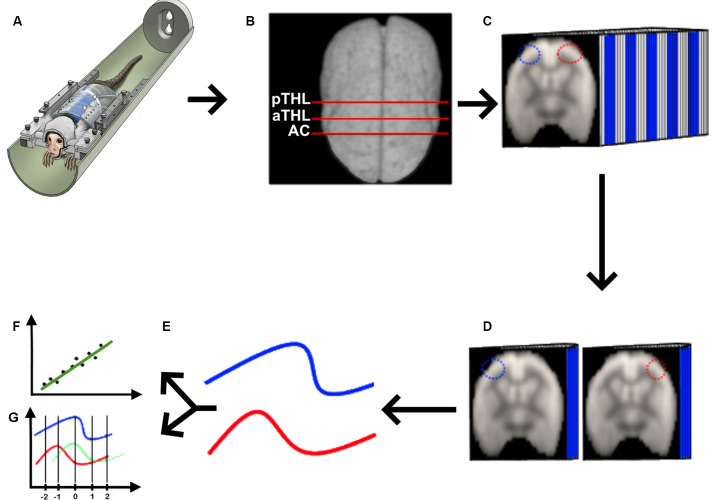
Quasi-periodic patterns (QPP) processing pipeline. **(A)** We acquired rs-fMRI data from awake marmosets (Liu et al., 2019), applied the standard rs-fMRI preprocessing pipeline described in “Materials and Methods” section, and **(B)** selected the coronal planes of the anterior commissure (AC), anterior thalamus (aTHL), and posterior thalamus (pTHL). **(C)** For each of the selected coronal planes, we drew homotopic ROIs (depicted as the blue and red ROIs), fed the 3D data into the QPP MATLAB script (Belloy et al., 2018; Abbas et al., 2019). Then, we extracted the QPP time series from each ROI **(D)**, and averaged them into 2D time courses **(E)**. These QPP time courses were used to calculate both the Pearson’s correlation **(F)** and the cross-correlation lag **(G)** in MATLAB.

**Figure 2:**
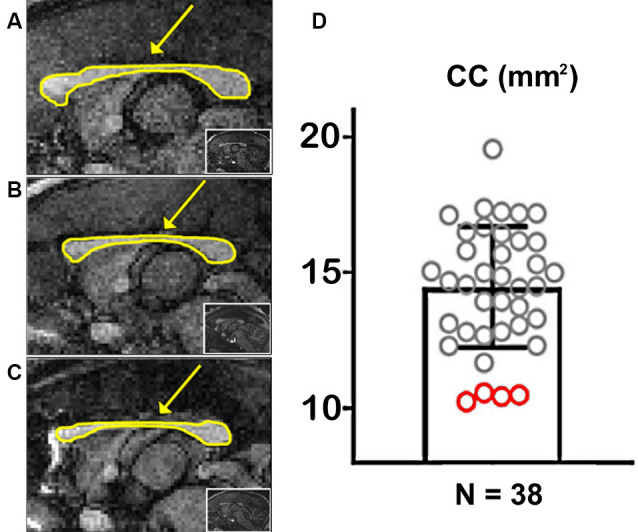
Marmoset populational Corpus Callosum (CC) quantification. **(A)** Example of a marmoset with a regular-sized CC. **(B,C)** Examples of two marmosets with a hypoplasic CC. The arrows point to the CC, outlined in yellow. The insets show whole-brain sagittal images. **(D)** Quantification of the CC area along the midline in 38 marmosets. Red circles portray CCD animals.

**Figure 3:**
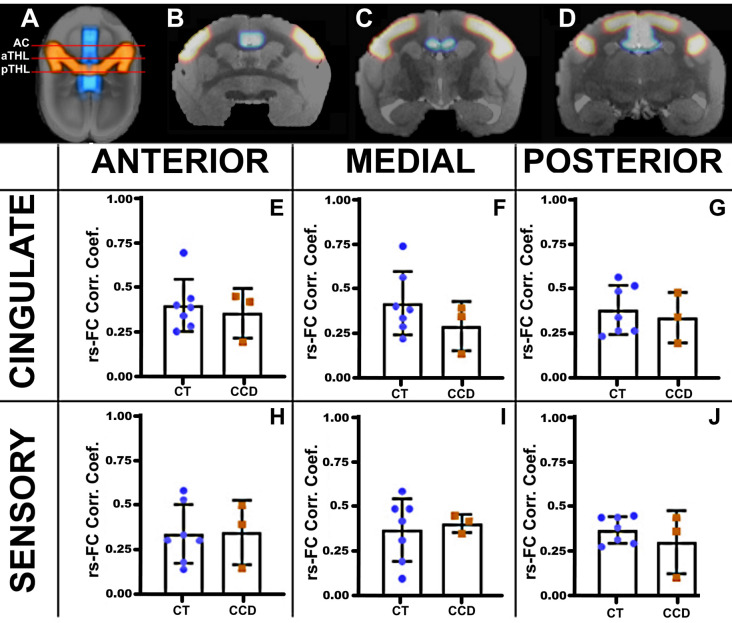
Marmoset resting-state functional correlation. **(A)** Axial view of a marmoset brain showing the regions of interest selected for analysis, the cingulate cortex (blue), and the sensory areas A1, S1, and S2 (red). Analyses were conducted in three coronal planes along the anteroposterior axis: the anterior plane at the level of the anterior commissure (AC; **B**), the medial plane at the level of the anterior thalamus (aTHL; **C**), and the posterior plane at the level of the posterior thalamus (pTHL; **D**). The plots show the strength of the resting-state functional connectivity (rsFC) for the homotopic cingulate cortex **(E–G)** and the sensory areas **(H–J)** in control (CT, blue dots) and CCD (red squares) animals. No differences in rsFC were found between control and CCD marmosets. Error bars = 1 st.dev.

For the human subjects, we registered the AAL atlas (AAL, RRID:SCR_003550, Tzourio-Mazoyer et al., 2002) to each individual and extracted the bilateral time series of the aCC, the pCC, and S1 as the representative interhemispheric cortical regions.

The FC coefficients were calculated in AFNI (Analysis of Functional NeuroImages, RRID:SCR_005927) by seeding and targeting both hemispheres of the different homotopic ROIs, described above, using the default clustering parameters.

#### QPP Processing

We acquired the awake rs-fMRI data (Figure 1A), standardized the 4D data of each animal in the time domain (Figure 1B), selected three coronal slices (at the level of the anterior commissure, anterior thalamus, and posterior thalamus as shown in Figure 1B), drew the homotopic ROIs for QPP seeds and ran the quasi-periodic-pattern (QPP) pipeline (Belloy et al., 2018; Abbas et al., 2019), as shown in Figure 1C. Briefly, the QPP pipeline uses a sliding window correlation method to find low-frequency repeating oscillation patterns. A template consisting of a 20-s window (10 images) is selected at the beginning of the scan and compared to the whole time series *via* a sliding correlation at each time point, and the resultant correlation coefficients are computed. The top 10 peak correlation values are used to generate an updated template. This process is iteratively repeated until there is a convergence of the spatiotemporal pattern, resulting in the final QPP (Belloy et al., 2018). Belloy et al. (2018) showed that 3,000-time points are sufficient to properly describe the QPP. As our marmosets’ scans have more than 3,000-time points each, we performed the QPP analysis in each animal. In contrast, because the human rsfMRI scans were shorter, we had to concatenate all rsfMRI runs obtained from the six AC subjects, the four HP subjects, and the five control subjects to calculate each group’s QPP, as previously performed (Abbas et al., 2019). We then used each of the homotopic templates (Figure 1D) to calculate the average QPP time course for each ROI (Figure 1E), from which we calculated the Pearson’s correlation between these signals (Figure 1F) and their cross-correlation delay (Figure 1G).

Group analysis for QPP differences was performed in Matlab (MATLAB, RRID:SCR_001622) using Pearson’s correlation of the homotopic QPP time series and *t*-tested for group differences. Additionally, we calculated the cross-correlation of homotopic QPPs to detect communication lags across hemispheres, computed the cross-correlation maxima, and compared between groups using *t*-tests.

## Results

### Marmoset Populational Study

We used the T_1_-weighted MRIs to calculate the CC’s midline cross-sectional area for each of the 38 marmosets (Figure 2). Figures 2A–C shows examples of cross-sectional areas for three marmosets. We found that four animals in our cohort had a significantly smaller CC cross-sectional area than the population average (Figure 2D). The reduced CC maintained all of their anatomical subdivisions in all of our animals. We found no other brain malformations in these animals, even malformations usually associated with CCD, such as colpocephaly and inversion of the cingulate gyrus (Tovar-Moll et al., 2007).

### Marmoset Functional Connectivity

We then paired three of these CCD animals with matching controls (standard sized CC) and performed awake rsfMRI. We calculated the awake resting-state functional connectivity (FC) between areas of the cingulate cortex and the sensory regions S1, S2, and A1, as investigated by previous groups (Belloy et al., 2018). We then compared the homotopic connections of the cingulate cortex and sensory areas that communicate through the CC at three anteroposterior levels (Figure 3). We found no unpaired *t*-test statistical differences between groups, suggesting that the CCD did not affect the homotopic strength of connectivity in the affected animals.

### Marmoset QPP

#### Marmoset Correlation

**Figure 4:**
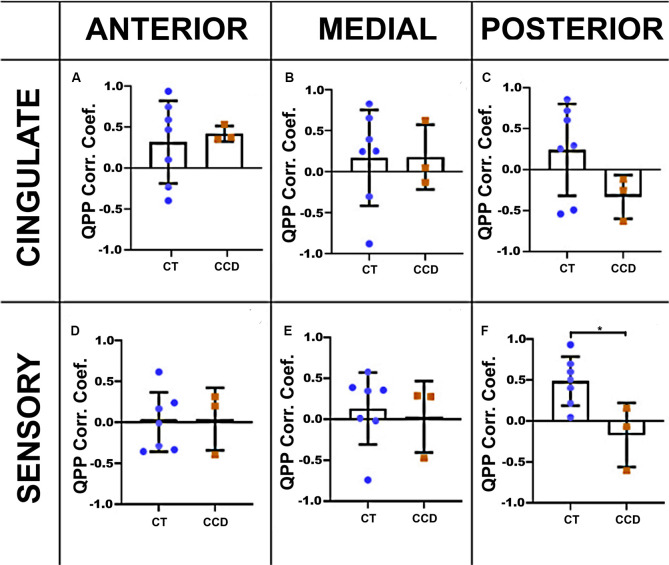
Marmoset QPP correlation. Inter-hemispheric QPP correlation coefficients of the cingulate cortex **(A–C)** and sensory regions **(D–F)** in the three coronal planes (anterior, medial, and posterior), respectively, as defined in Figure 3. There was a significant reduction in the inter-hemispheric QPP correlation in the sensory areas of CCD animals compared to controls (CT) at the posterior level **(F)**, *p = 0.02. Error bars = 1 std.dev.

We calculated the cortical QPP of low-frequency oscillations to explore further the dynamic rsFC between homotopic regions in the cingulate cortex and sensory areas in these animals. We extracted the time series of both hemispheres in the cingulate cortex and sensory brain regions in the three coronal planes. The correlation coefficient of the CCD animals was lower than the Control group (unpaired *t*-test *p* = 0.019) in the sensory areas at the posterior level (Figure 4F), but not at the anterior or medial levels (Figures 4D,E). The cingulate cortical regions were not affected in the CCD animals (Figures 4A–C).

#### Marmoset Cross-correlation

**Figure 5:**
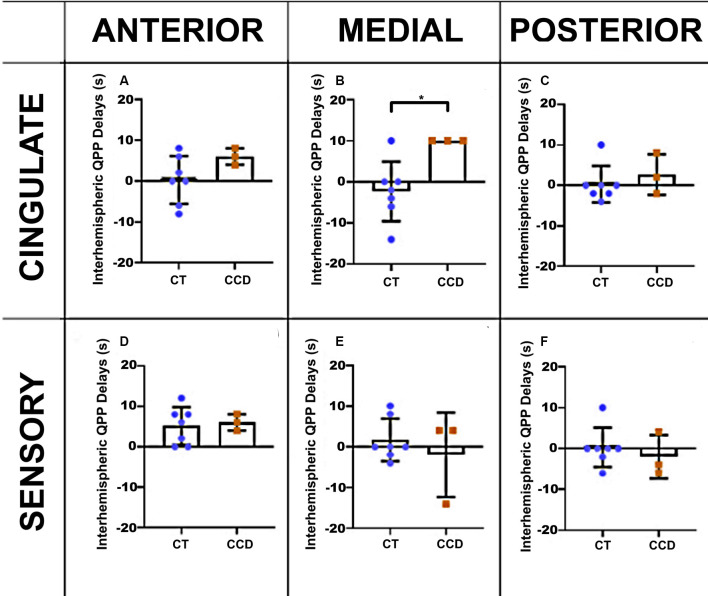
Marmoset QPP cross-correlation. Inter-hemispheric QPP cross-correlation coefficients of the cingulate cortex **(A–C)** and sensory regions **(D–F)** in three coronal planes (anterior, medial, and posterior), respectively, as defined in Figure 3. There was a significant reduction in inter-hemispheric delay in the cingulate cortex of CCD animals compared to controls (CT) at the medial level, *p = 0.02. Error bars = 1 std.dev.

To further understand the relationship between the altered structure and its impact on brain function, we also analyzed the correlation lag (cross-correlation) between the same brain areas in the three anteroposterior planes. We found that the cingulate cortex in the CCD animals has a delay of ~10 s between brain hemispheres at the medial level (unpaired *t*-test *p*-value = 0.02, Figure 5B) but not at the anterior or posterior levels (Figures 5A,C). The sensory areas showed no correlation lags between hemispheres (Figures 5D–F). Supplementary Figure 1 shows that the cross-correlation delay is specific to the QPP time courses and not to the conventional rsFC signal.

### Human rs-fMRI

**Figure 6:**
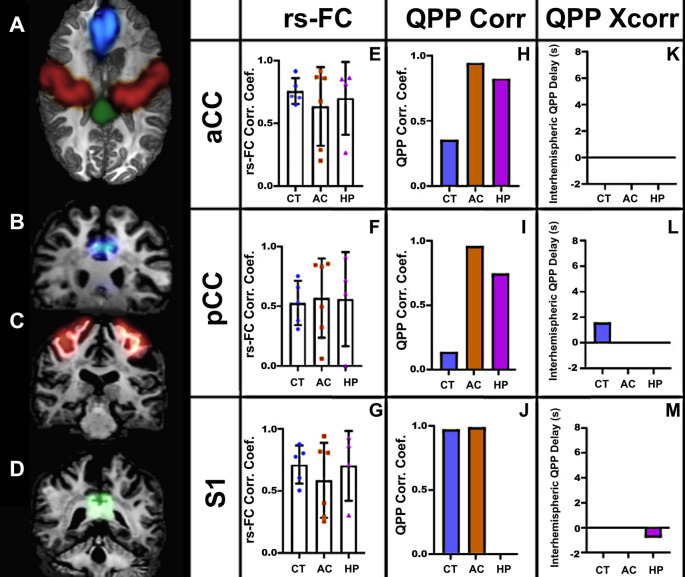
Human rs-fMRI. **(A)** Axial view of a human brain showing the regions of interest selected for analysis: aCC (blue), S1 (red), and pCC (green). Coronal views of the aCC **(B)**, S1 **(C)**, and pCC **(D)**. The resting-state correlation coefficients **(E–G)**, QPP correlation coefficients **(H–J)**, and interhemispheric delays **(K–M)** in three groups: agenesic (AC), hypoplasic (HP), and controls (CT). Error bars = 1 std.dev.

We performed similar analyses in human subjects (Figure 6). As in marmosets, the strength of rsFC in CCD patients did not differ from controls, even with the large callosal structural changes (Figures 6E–M). However, the QPP correlation analysis showed clear differences between CCD patients and controls, both for the cingulate cortex and sensory area. In the cingulate cortex, CCD patients presented high QPP correlation coefficients at both anterior and posterior levels (Figures 6H–I) relative to controls. However, in the sensory area, AC patients, like controls, show a high QPP correlation, whereas HP patients had no interhemispheric QPP correlation (Figure 6J). This difference between CCD patients can be partially explained by the interhemispheric QPP delay shown by HP patients (Figure 6M). Supplementary Figure 2 shows that the cross-correlation delay is specific to the QPP time courses and not to the conventional rsFC signal.

## Discussion

The development of the CC starts at the aCC with the pioneer axons (the first axonal fibers to cross the midline), followed by a small expansion in the anterior direction to form the genu, and later by a large expansion in the posterior direction to form the body and splenium (Donahoo and Richards, 2009). Simultaneously to the development of the CC along the anteroposterior axis, interhemispheric axonal fibers project along the mediolateral axis to connect homotopic cortical regions. To understand whether these developmental gradients along the anteroposterior and mediolateral axes affect the interhemispheric connectivity, we decided to analyze the rsFC data from medial (aCC and pCC) and lateral (sensory) in CCD marmosets and humans at three different coronal planes along the anteroposterior axis.

We know that CCD has a very intimate anatomical relationship with the midline (Paul et al., 2007; Paul, 2011). We also know that global cortical connectivity is significantly altered in CCD patients (Owen et al., 2013a,b; Tovar-Moll et al., 2014; Jakab et al., 2015; Lazarev et al., 2016). The anterior portion of the cingulate cortex is involved in the development of the CC, and the source of the first axons to cross the midline, the pioneer axons (Donahoo and Richards, 2009). The posterior cingulate cortex, one of the major hubs of the DMN (Liu et al., 2019), is immediately adjacent to the midline. We also analyzed the more lateral sensorimotor areas of the cortex, which project homotopically to the other hemisphere *via* the CC (follower axons).

We found that these homotopic areas of the brain were still functionally and robustly connected both in CCD marmosets (Figure 3) and humans (Figure 6), as there were no differences in rsFC between CCD and controls, in agreement with previous reports of strong bilateral communication in subjects with different presentations of CCD (Tyszka et al., 2011). Furthermore, studies investigating the functional connectome in CCD patients found no differences in network-based-statistics relative to healthy subjects (Owen et al., 2013a). This was true even for AC subjects that had a total absence of callosal fibers connecting the two hemispheres (Tovar-Moll et al., 2014). One possible explanation is that these patients showed alternative fiber pathways connecting the two hemispheres (Tovar-Moll et al., 2014).

Callosal fibers regulate the lateralization of brain function dynamically *via* a balance of inhibitory and excitatory signaling to specific cortical regions to synchronize interhemispheric brain activity (Hinkley et al., 2012). To understand whether the presence of CCD affected interhemispheric synchronization between homotopic regions, we resorted to computing and analyzing the QPP of low-frequency oscillations in both networks (Belloy et al., 2018). The QPP is a powerful method to evaluate dynamic variations in brain states and can be employed to investigate interhemispheric interactions.

Relative to controls, CCD marmosets had the same QPP correlation coefficient in all three anteroposterior levels of the cingulate cortex. However, the QPP correlation coefficient for the posterior level of the sensory areas was significantly smaller in CCD animals than in controls (Figure 4F). The same finding was valid for HP (but not AC) human subjects (Figure 6J). These results suggest that hypoplasia of the CC compromises the ability of homotopic regions to communicate synchronously. These findings are consistent with the delayed development of the CC, which would affect the posterior and lateral regions of the cortex. They may also help explain why the CCD human subjects had a high QPP correlation at the anterior and medial levels of the CC relative to healthy subjects (Figures 6H,I), as healthy subjects presumably have a higher number of connections through the anterior and medial planes relative to the posterior plane. The increase in low-frequency connectivity of CCD patients is associated with an interhemispheric delay of the healthy subjects at the pCC level (Figure 6L). It suggests that the altered interhemispheric connections due to the malformations of the CC connect the cingulate cortex in a highly efficient manner that can maintain the low-frequency (but not the high-frequency) synchronization between hemispheres.

Interestingly, the CCD marmosets presented an elevated interhemispheric QPP lag in both the anterior (N.S.) and the medial planes of the cingulate cortex (Figures 5A,B). Because the QPP correlation was normal at both levels (Figures 4A,B), these animals seem to maintain synchrony of interhemispheric communication in the cingulate cortex, albeit with a delay. We did not observe similar findings in human CCD subjects.

Collectively, our results show that CC malformations impact the interhemispheric lag of the cingulate cortex and the synchrony of sensory regions at low frequencies. Our results also show that this interference is not homogeneous along with the anteroposterior and mediolateral directions, and follows the developmental gradients of the CC. Our study shines a light on how the hypoplasic CC misconnects homotopic regions and opens an essential line of research to explain the biological causes of the symptoms exhibited by CCD patients and how to mitigate them. Additionally, we show how marmosets can be used as a model of callosal malformations to close the translational gap in CCD research. In future work, we intend to use ultra-high-resolution diffusion MRI (Liu et al., 2019) to map callosal connections in CCD marmosets and perform rigorous assessments of behavior that may help explain the relationship between the callosal malformations and the learning and behavior symptoms commonly found in human CCD patients.

## Data Availability Statement

The raw data supporting the conclusions of this article are available from the corresponding author upon reasonable request.

## Ethics Statement

The animal study was reviewed and approved by the Animal Care and Use Committee of the National Institute of Neurological Disorders and Stroke. The studies involving human participants were reviewed and approved by the Ethics committee of the D’Or Institute for Research and Education (Rio de Janeiro, Brazil). Written informed consent to participate in this study was provided by the participants’ legal guardian/next of kin. Written informed consent was obtained from the individual(s), and minor(s)’ legal guardian/next of kin, for the publication of any potentially identifiable images or data included in this article.

## Author Contributions

DS, CY, CL, and XT acquired animal data. DS and ACS designed experiments and analyses. FT-M provided human CCD data. DS, ACS, FT-M, and RL wrote the manuscript, and all authors contributed with the manuscript revision. All authors contributed to the article and approved the submitted version.

## Conflict of Interest

The authors declare that the research was conducted in the absence of any commercial or financial relationships that could be construed as a potential conflict of interest.
