# Supplementary material for: Dynamic Interhemispheric Desynchronization in Marmosets and Humans With Disorders of the Corpus Callosum
Source: Front Neural Circuits. 2020 Dec 21;14:612595. doi: 10.3389/fncir.2020.612595 (PMC7779638; doi:10.3389/fncir.2020.612595)
Supplement: Supplementary file 1 [file Data_Sheet_1.docx]

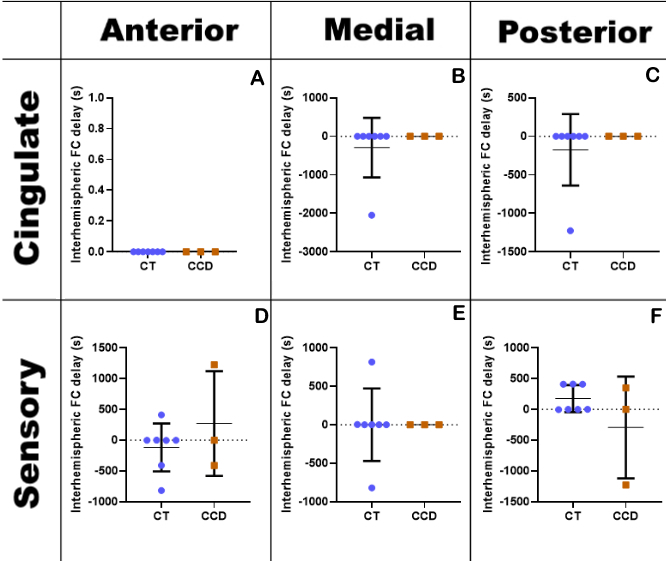


**Supplementary Figure 1. Marmoset rs-fMRI interhemispheric delays.** Inter-hemispheric resting-state cross-correlation coefficients of the cingulate cortex **(A-C)** and sensory regions **(D-F)** in three different anatomical planes (anterior, medial, and posterior) defined in **Figure 3**. Error bars = 1 std.dev.


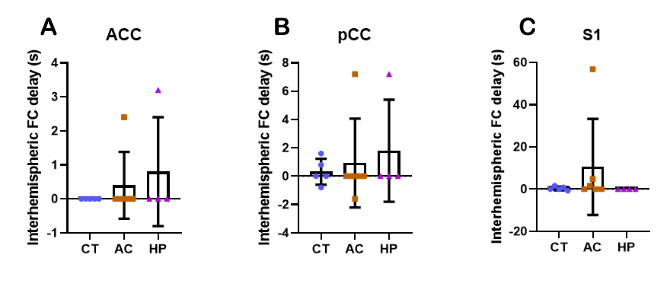


**Supplementary Figure 2. Human rs-fMRI interhemispheric delay.** Inter-hemispheric resting-state cross-correlation coefficients of the anterior cingulate cortex (aCC) **(A)**, posterior cingulate cortex (pCC) **(B)**, and primary somatosensory cortex (S1) **(C)** defined in **Figure 6**. Error bars = 1 std.dev.
